# *T. gondii* rhoptry protein ROP18 induces apoptosis of neural cells *via* endoplasmic reticulum stress pathway

**DOI:** 10.1186/s13071-015-1103-z

**Published:** 2015-10-21

**Authors:** Lijuan Wan, Lingli Gong, Wei Wang, Ran An, Meijuan Zheng, Zongru Jiang, Yuewen Tang, Yihua Zhang, He Chen, Li Yu, Jilong Shen, Jian Du

**Affiliations:** Distinguished Young Scholar of Anhui Province. Department of Biochemistry and Molecular Biology, School of Basic Medical Sciences, Anhui Medical University, No.81 Meishan Road, Anhui P.O. Box 71, Hefei, 230032 China; Department of Parasitology, School of Basic Medical Sciences, Anhui Medical University, Hefei, China; Clinical Laboratory, the First Affiliated Hospital, Anhui Medical University, Hefei, Anhui China; The Key Laboratory of Zoonoses and Pathogen Biology Anhui, Hefei, China; Department of Microbiology, Anhui Medical University, Hefei, China

**Keywords:** *T. gondii*, ROP18, ER stress, Apoptosis

## Abstract

**Background:**

The neurotropic parasite *T. gondii* is widespread among mammalian hosts including humans. During the course of *T. gondii* infection, the central nervous system is the most commonly damaged of all invasive organs. The polymorphic rhoptry protein ROP18 has been identified as a key factor in the pathogenesis of *T. gondii*; however, the molecular mechanism by which this protein exerts neuropathogenesis remains elusive.

**Methods:**

Immunofluorescence staining was performed to detect neuropathogenesis of the mouse brain tissues. The apoptosis of neural cells and the expressions of related proteins in the endoplasmic reticulum stress (ER Stress)-mediated apoptosis pathway were detected by flow cytometry and Western blotting.

**Results:**

Immunofluorescence staining reveals induction of the propidium iodide (PI) - positive neural cells in mouse cerebral cortex and hippocampus infected with ROP18 over-expressing transgenic tachyzoites. Western blotting analyses reveal that ROP18 increases the expressions of cleaved caspase-12, CHOP and cleaved caspase-3 when compared to the control groups. After the pretreatment of Z-ATAD-FMK (a specific caspase-12 inhibitor), the apoptotic level of neural cells had an apparent decline, and correspondingly, the expressions of those related proteins were notably decreased.

**Conclusions:**

Our findings here highlight that the virulence factor ROP18 in *T. gondii* may contribute to neuronal apoptosis through the ER stress-mediated apoptosis pathway, which may be a potential molecular mechanism responsible for neurological disorders of toxoplasmosis.

## Background

*T. gondii* is a ubiquitous obligate intracellular parasite infecting a wide range of mammalian hosts including humans [1–3]. Infections with *T. gondii* are generally subclinical in healthy individuals, but toxoplasmosis remains a major problem in immunosuppressed adults or developing fetuses [4]. In patients with severe immune dysfunction, a reactivation of the infection produces the neurological manifestations or even fatal Toxoplasmic encephalitis (TE) due to reactivation of Toxoplasma cysts in the brain [5]. For those patients, dormant encysted bradyzoites can reactivate into fast replicating tachyzoites and cause severe damage to the brain [6]. TE is the most vital outcome of toxoplasmosis in immunosuppressed individuals and the main symptoms including focal seizures, cranial nerve disturbances, altered mental state, ataxia, sensory abnormalities, hemiparesis, meningismus and neuropsychiatric disorders as well [2].

Although toxoplasmosis can be associated with multi-organ involvement, the central nervous system (CNS) is the most commonly affected of all invasive organs [7, 8]. As a neurotropic parasite, little is known about which effectors of the type I RH strain elicit neuropathogenesis during infection [7, 9]. Previous work revealed a polymorphic, parasite rhoptry protein, known as ROP18, is a key virulence determinant among different *T. gondii* clonal lineages [10–13]. The recent results have shown that ROP18 has several targets in the host cell, including IRGs (immunity-related GTPases) [14, 15] and NF-κB p65 [16]. IRGs are strongly induced by interferon-γ (IFN-γ) and are important in the innate immune response against *T. gondii* [14]. ROP18 contributes to avoidance of IRG recruitment to the parasitophorous vacuole membrane (PVM), thus protecting the parasite from clearance in interferon-activated macrophages. The nuclear factor NF-κB transcription factor has essential roles in immune and inflammatory responses. Its p65 subunit is regulated by several post-translational modifications, including phosphorylation, acetylation and ubiquitination. Our recent results show that ROP18 phosphorylates the host p65 and targets this protein to the ubiquitin-dependent degradation, thus inhibiting the NF-κB pathway in infected macrophages [16].

Our previous study also revealed that both the canonical type I RH strain and TgCtwh3, a representative strain prevalent in China, can induce the apoptosis of neural stem cells through ER stress signaling pathways [17, 18]. In this study, we investigated the effect of rhoptry protein ROP18 on the apoptosis of neural cells. Our results presented here showed that ROP18 can stimulate neural cell death through inducing ER stress-mediated apoptosis pathway.

## Methods

### Ethical statement

The study protocol was approved from the Institutional Review Board of the Institute of Biomedicine at Anhui Medical University (Permit Number AMU 26–093628), which records and regulates all research activities in the school. All surgeries were performed under anesthesia, and all efforts were made to minimize animal suffering.

### Parasite and cell lines

ROP18 over-expressing transgenic RH strain (ROP18-RH) was constructed as previously described. Briefly, the 5′-UTR-TUB promoter-ROP18 -Ty1-HXGPRT-3′ -UTR fragment was transfected into the *Δku80Δhxgprt* RH strain parasites (kindly provided by Professor John C. Boothroyd, Stanford University, USA) by electroporation. Stable integrants were selected in media with 50 μg/ml mycophenolic acid and 50 μg/ml xanthine and cloned by limiting dilution. Construction of ROP18-RH strain was confirmed by PCR, IF and Western-blotting [16]. Then the parasites of ROP18-RH strain were harvested from the mouse peritoneal exudates by injection on the third day after infection, and were isolated by centrifugation to discard the contaminating host cells. Then the parasites were maintained by serial passage in the mouse N2A cells (ATCC, Neuro2a) for further experiments. The mouse neuroblastoma Neuro2a (N2a) cells were cultured in DMEM supplemented with 20 % FBS and 1 % penicillin/streptomycin in a humidified 5 % CO_2_ atmosphere. All parasite strains and cell lines were routinely assessed for mycoplasma contamination, and no contamination was detected.

### Plasmid and reagents

Amplification of the open reading frame encoding *T. gondii* ROP18 (GenBank ID: AM075204.1) was achieved through RT-PCR of the RH tachyzoite RNA. Caspase-12, CHOP and caspase-3 antibodies were purchased from Cell Signaling Technology (USA); GAPDH antibody was purchased from Santa Cruz (USA).

### Immunofluorescence

Brain sections were hydrated and rinsed in PBS. Then they were placed in ACSF (artificial cerebrospinal fluid) containing 5 μg/ml PI for 30 min. After antigen retrieval, the brain sections were premeabilized, then incubated with primary antibody at 4 °C overnight. FITC-conjugated goat anti-mouse IgG, rhodamine-conjugated goat anti-rabbit IgG and DAPI dye were used for antigen and DNA visualization. The images were captured using a fluorescent microscopy (Olympus BX60, Tokyo, Japan).

### Detection of apoptosis

The apoptosis of N2a cells were determined following the instruction of Annexin V-PE/7-AAD kit (BD, USA). Briefly, the N2a cells were harvested, then washed twice with cold PBS, and incubated in 1 × binding buffer (10 mM HEPES, 0.14 M NaCl, and 0.25 mM CaCl_2_) containing PE Annexin V and 7-AAD for 15 min. Then stained cells were analyzed using a Faces Calibur flow cytometer (BD Biosciences, USA) within 1 h, and the data were analyzed using FCS Express 4.0 software. Annexin V-PE+/7-AAD − cells represent the early apoptotic cells, and annexinV-PE+/7-AAD+ cells represent the late apoptotic cells.

### Western blotting analysis

To further identify the apoptosis of N2a cells, the expressions of caspase-12, CHOP and caspase-3 were determined using Western blotting analysis. Western blotting was conducted as described previously [16]. Briefly, after the N2a cells were infected with ROP18-RH or RH tachyzoites at an m.o.i of ~3 for 24 h, they were harvested, washed with PBS, and lysed in a lysis buffer (50 mM HEPES, pH7.4, 150 mM NaCl, 2 mM EGTA, 1 % Triton X-100, 1 mM phenylmethylsulfonyl fluoride, 10 g/ml leupeptin, and 10 g/ml pepstatin A) containing a protease inhibitor mixture (Sigma, USA). The cell lysates were separated through SDS-PAGE, transferred to nitrocellulose membranes, probed with the corresponding antibodies, and developed using an ECL kit.

### Statistical analysis

All quantitative results were expressed as mean ± SD. Statistical analysis of variance used was a two-way ANOVA followed by the Scheffe’s test. *P* < 0.05 was considered statistically significant.

## Results

### ROP18 induced the apoptosis of neural cells *in vivo*

**Fig. 1:**
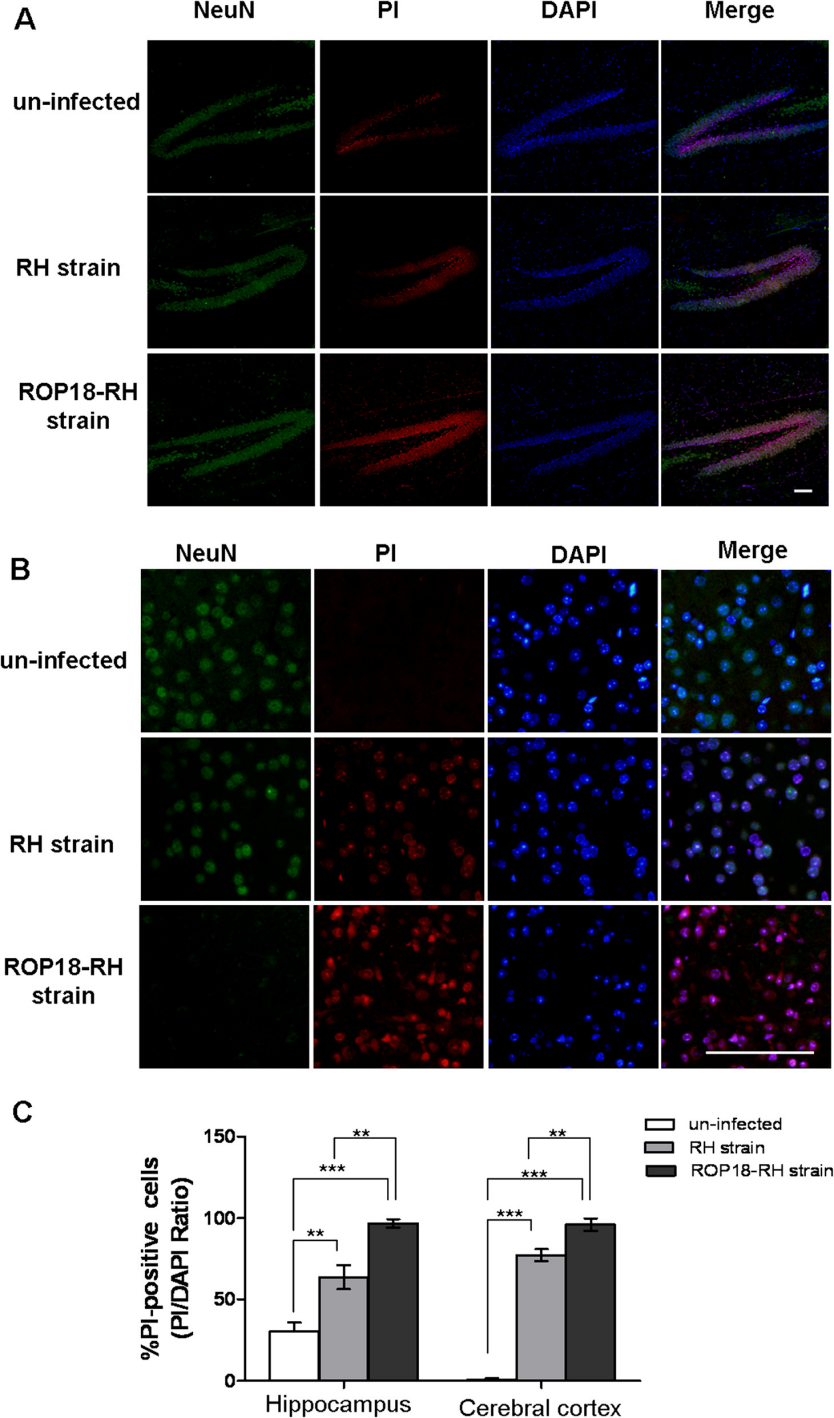
Immunofluorescence analysis of the brain from mice infected with over-expressing ROP18 transgenic parasites. Brain sections from animal groups infected with RH or ROP18-RH tachyzoites were double stained with NeuN (a specific marker of neuron cell) and PI (a marker of cell death). **a** the cerebral cortex; (**b**) hippocampus. NeuN (green); PI (red); DAPI (blue) was used to stain the nuclei. Scale bar = 50 μm; (**c**) The quantitative data of panel (**a**) and (**b**). Values are expressed as mean ± SD on three individuals. **P < 0.01; ***P < 0.001 vs. un-infected control

To assay the effects of ROP18 on the survival of neurons during infection, we infected *BALB/c* mice intraperitoneally with 1000 tachyzoites from ROP18 over-expressing parasites (ROP18-RH) or RH tachyzoites [16]. Pathology in the brain tissues of animals was examined as soon as obvious clinical manifestations were observed. Immunofluorescent staining NeuN (a specific neuron marker) and propidium iodide (PI) staining were used to reveal the neuronal damage. We found that in the un-infected group, the cerebral cortex was intact and there were many neurons there. However, the loss of neurons in the hippocampus of the mice infected with ROP18-RH or RH tachyzoites were 96.82 ± 2.62 % and 63.74 ± 7.38 %, significantly higher than those in the un-infected group (30.24 ± 5.2 %), accordingly (**P < 0.001, ***P < 0.0001). Consistently, the loss of neurons in the cerebral cortex of the mice infected with ROP18-RH or RH tachyzoites were 96.05 ± 3.92 and 77.28 ± 3.67 %, significantly higher than those in the un-infected group (1.88 ± 1.08 %), accordingly (***P < 0.0001). In addition, the numbers of dead neural cells in the cerebral cortex as well as in the hippocampus of the mice infected with ROP18-RH tachyzoites were significantly higher than RH tachyzoites infected group, accordingly (**P < 0.001) (Fig. 1). The results suggest ROP18 may induce the loss of neurons during infection *in vivo.*

### ROP18 induced the apoptosis of neural cells *in vitro*

**Fig. 2:**
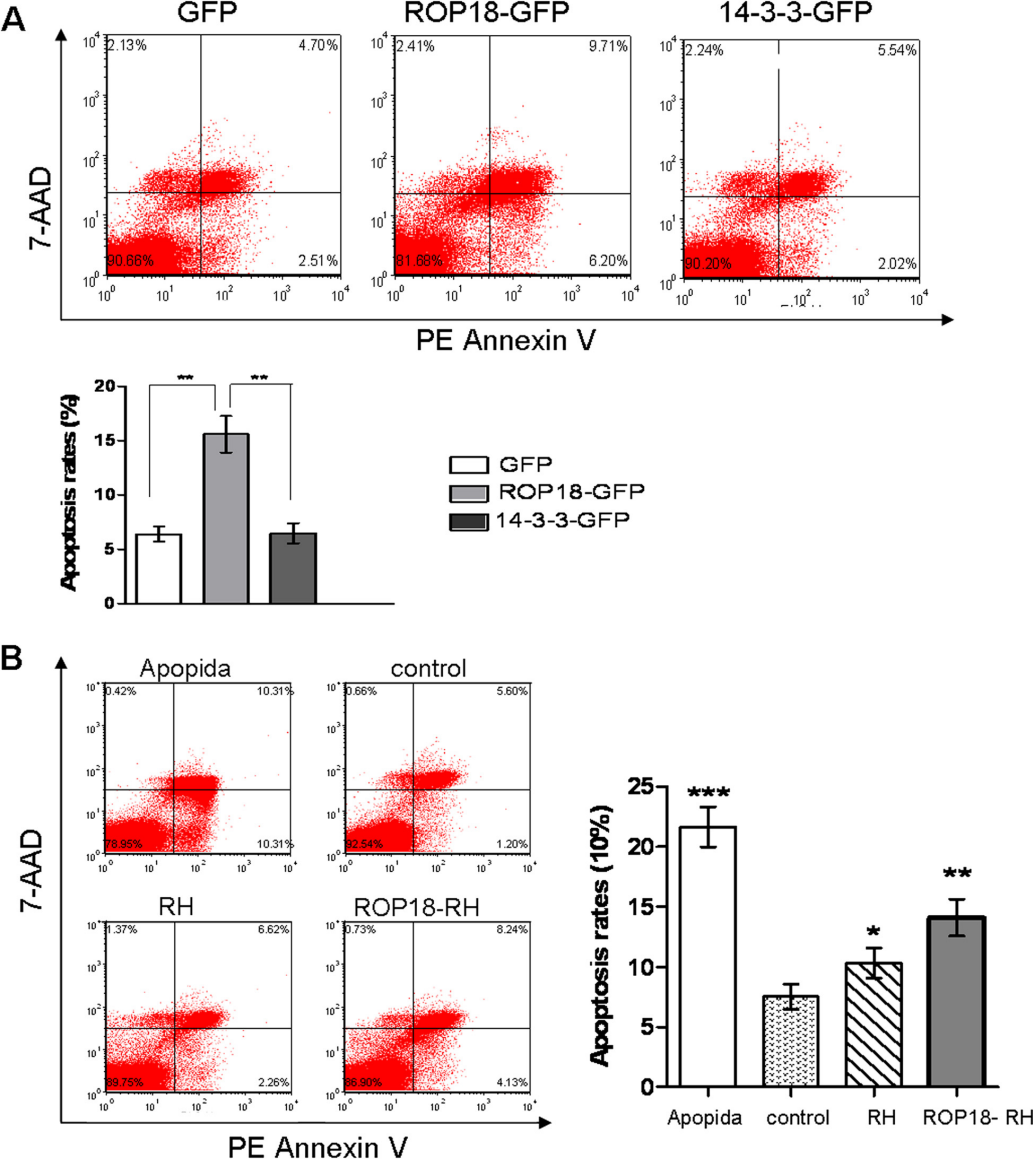
ROP18 induced the apoptosis of N2a cells. **a**The N2a cells were transfected with ROP18-GFP, 14-3-3-GFP or control GFP vector for 24 h. Then the apoptosis of the cells were detected by flow cytometry after Annexin V-PE/7-AAD staining. **b** The N2a cells were infected with ROP18-RH, RH tachyzoites at an m.o.i of ~3 or treated with Apopida (apoptosis inducer A) for 24 h. Then the apoptosis of the cells were detected by flow cytometry after Annexin V-PE/7-AAD staining. The plots are from a representative measurement and the quantitative data were expressed as mean ± SD on three different assays (*n* = 3). *P < 0.05; **P < 0.01; * **P < 0.001 vs. Negative controls

To investigate if the observed cell death of neurons from the mice infected with ROP18-RH or RH tachyzoites result from apoptosis, we performed FCM analysis with Annexin V-PE/7-AAD staining in a double-fluorescence assay. The mouse neuroblastoma line N2a cells were transfected with expression vectors for ROP18 or another parasite protein present in the parasitophorous vacuole, 14-3-3. We then ectopically expressed these parasite proteins and analyzed the effects on the apoptosis of N2a cells. Strikingly, the over-expression of ROP18, but not 14-3-3, in N2a cells led to high levels of apoptosis (Fig. 2a)*.* To investigate this further, we infected N2a cells with ROP18-RH or RH tachyzoites. FCM analysis showed that ROP18-RH, compared with RH tachyzoites, obviously enhanced the apoptosis of neural cells (Fig. 2b). Collectively, these results provide that ROP18 induced the apoptosis of neural cells *in vitro.*

### ROP18 induced the apoptosis of neural cells *via* ER stress pathway

**Fig. 3:**
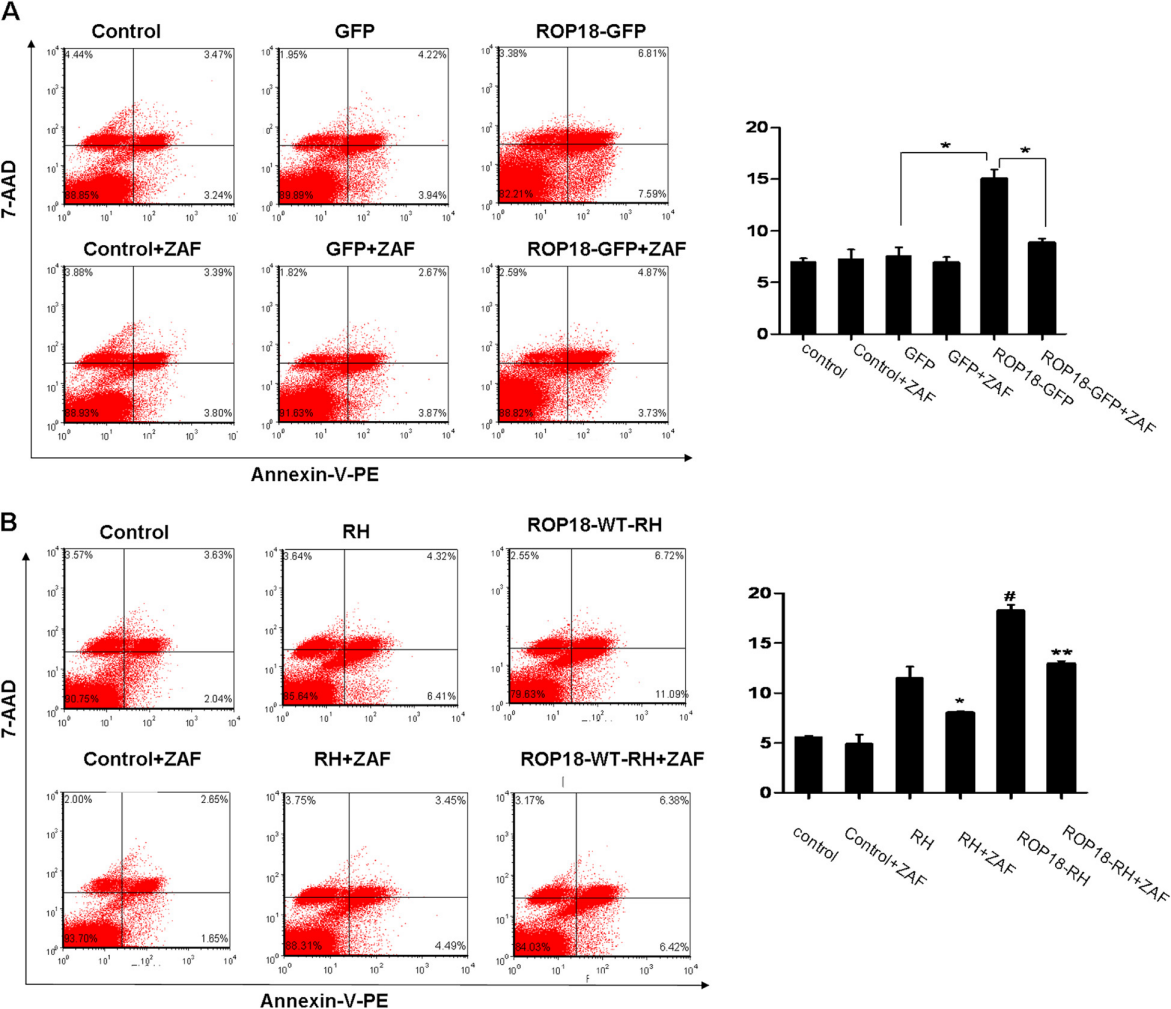
Analyses of inhibitors on the apoptosis levels of N2a cells. **a** After the N2a cells were pretreated with or without Z-ATAD-FMK (ZAF) for 6 h, they were transfected with ROP18-GFP or control GFP vector for 24 h. Then the apoptosis of the cells were detected by flow cytometry after Annexin V-PE/7-AAD staining. ROP18-GFP or GFP+ ZAF stands for the N2a cells pretreated with ZAF, and then transfected with either ROP18-GFP or control GFP vector. The plots are from a representative measurement and the quantitative data were expressed as mean ± SD on three different assays (*n* = 3).*P < 0.05, compared with the controls. **b** After the N2a cells were treated with or without ZAF for 6 h, they were infected with ROP18-RH or RH tachyzoites at an m.o.i of ~3 for 24 h. Then the apoptosis of the cells were detected by flow cytometry after Annexin V-PE/7-AAD staining. ROP18-RH + ZAF or RH + ZAF stands for the N2a cells pretreated with ZAF, and then infected with either ROP18-RH or RH tachyzoites. The plots are from a representative measurement and the quantitative data were expressed as mean ± SD on three different assays (*n* = 3).*P < 0.05 vs. RH group; **P < 0.01 vs. ROP18-RH group; ^#^ *P* < 0.05 vs. RH group

**Fig. 4:**
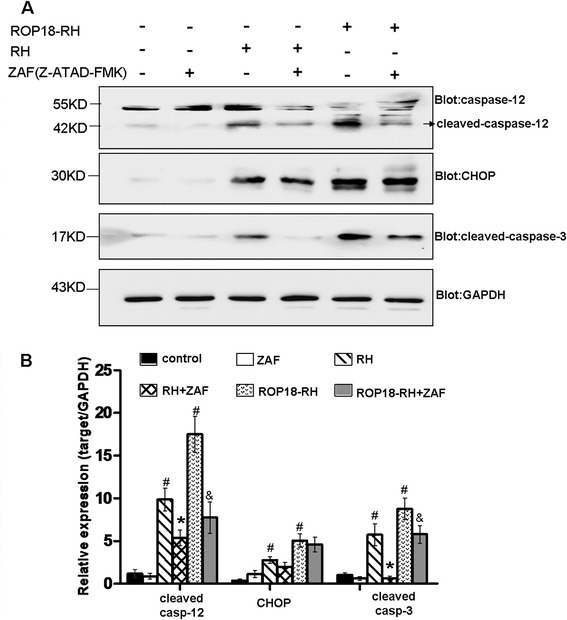
Western blotting analyses of inhibitors on caspase-12, caspase-3 and CHOP expression levels in N2a cells. After the N2a cells were treated with or without ZAF, they were infected with ROP18-RH or RH tachyzoites at an m.o.i of ~3 for 24 h. **a** The presented figures are from a representative study and the quantitative data were expressed as mean ± SD on different assays (*n* = 3). The N2a cells without the infection of tachyzoites or only pretreated with ZAF were used as controls. ROP18-RH + ZAF or RH + ZAF stands for the N2a cells pretreated with ZAF, and then infected with either ROP18-RH or RH tachyzoites. **b** The quantitative data of **a** panel. The experiments were repeated three times (*n* = 3). ^#^ *P* < 0.001 vs. control; * *P* < 0.05 vs. RH group; & *P* < 0.05 vs. ROP18-RH group

Our previous results have shown that both the RH and TgCtwh3 strains can induce the apoptosis of neural stem cells through ER stress signaling pathways [17, 18]. To examine whether ROP18 was the effective molecule involved in the ER Stress-mediated apoptosis pathway, N2a cells transfected with ROP18-GFP or GFP vector were treated with 5 μmol/L Z-ATAD-FMK for 6 h and FCM analysis was performed. Consistently, the apoptotic rates of cells transfected with ROP18-GFP were significantly higher than the control groups (Fig. 3a). After treatment with Z-ATAD-FMK, the apoptotic rates of the cells transfected with ROP18-GFP were 8.87 ± 0.38 %, significantly lower than those in the Z-ATAD-FMK untreated ROP18-GFP group (15.04 ± 0.91 %), accordingly (*P < 0.05) (Fig. 3a). To further investigate it, the cells infected with ROP18-RH or RH tachyzoites were treated with 5 μmol/L Z-ATAD-FMK for 6 h. After treatment with Z-ATAD-FMK, the apoptotic rates of cells infected with ROP18-RH or RH tachyzoites were 12.98 ± 0.25 % and 8.05 ± 0.15 %, significantly lower than those in the Z-ATAD-FMK untreated ROP18-RH group (18.25 ± 0.62 %) and untreated RH group (11.53 ± 1.13 %), accordingly (*P < 0.05, **P < 0.01) (Fig. 3b). Consistently, the apoptotic rates of the cells infected with ROP18-RH tachyzoites (18.25 ± 0.62 %) were significantly higher than RH tachyzoites infected group (11.53 ± 1.13 %) (Fig. 3b). Immunoblotting analysis further displays that the expression levels of cleaved caspase-12, CHOP and cleaved caspase-3 in the N2a cells increased significantly in both the ROP18-RH and RH tachyzoites infected groups when compared to the control group. When the cells were pretreated with a caspase-12 inhibitor, Z-ATAD-FMK, the expressions of cleaved caspase-12 and caspase-3 in the inhibitor-pretreated groups significantly decreased (Fig. 4) whereas no significant differences were detected in the levels of CHOP when they were compared to the control groups.

## Discussion

Life-threatening toxoplasmic encephalitis is a common opportunistic infection of the CNS that usually results from reactivation of latent cysts into tachyzoites in the brain tissue of immunosuppressed individuals [19, 20]. After reaching the CNS, the parasites can invade all nucleated cells and initiate activation of resident microglia and astrocytes [21–23]. In the brain tissues, microglial cells, astrocytes and neurons are all susceptible to *T. gondii* infection [2, 24]. Microglia cells are implicated as essential mediators of the brain’s immune response to injury, inflammation or the presence of pathogens [25, 26]. During the course of *T. gondii* infection, microglia cells can secrete pro-inflammatory cytokines and lead to the neuronal cell damage of inflammatory surroundings [27]. However, neurons are fundamental elements of the CNS, we hypothesized that there may be the possible direct mechanisms of *T. gondii* on neurons to disrupt their survival and function [2]. Accumulated studies conducted in different animal experimental models have found that behavioural changes occur upon acute and chronic *T. gondii* infection [28]. One of the most ‘convenient’ explanations for the neuropsychological and behavioural deficits could be that the parasites directly inject effective molecules into the neurons interfering with their activity and function [2].

As noted, ROP18 is a Ser/Thr kinase related to the ROP2 family, secreted from rhoptry organelles and relocalized to the PVM during invasion [14, 16, 29, 30]. During host cell invasion, ROP18 is secreted to the PVM where it is tethered to the cytosolic face of this host-pathogen interface. They are thought to modify the host cell environment, thus favoring intracellular parasitism [14]. Although the functional role of rhoptry proteins in the biology of *T. gondii* has not been clearly elucidated, numerous studies have highlighted the role of ROP18 as the key virulence factor in the pathogenesis of *T. gondii* infection [10, 11, 31]. Indeed, we have previously demonstrated that ROP18 kinase activity is responsible for the phosphorylation and degradation of p65, thereby down-regulating Th1 responses and causing M2 phenotypes in macrophage [16]. Here, we report that ROP18 is involved in the apoptosis of neural cells.

ER stress conditions have been observed in numerous diseases including ischemia/reperfusion injury, neurodegeneration, as well as infectious diseases [32–35]. Conditions that interfere with ER function lead to the accumulation and aggregation of unfolded proteins. ER trans-membrane receptors detect the onset of ER stress and initiate the unfolded protein response (UPR) to restore normal ER function. In eukaryotic cells, three ER trans-membrane proteins mediate the canonical UPR: the two kinases, IRE1 (inositol requiring enzyme 1) and PERK (PKR-like eukaryotic initiation factor 2a kinase), and the transcription factor precursor ATF6 (activating transcription factor 6). If the stress is prolonged, or the adaptive response fails, apoptotic cell death ensues through activation of caspase-12, CHOP and/or JNK (c-JUN NH2-terminal kinase) [36–38]. Many of these changes are induced by intracellular pathogens, so it is not surprising that ER stress is induced by cellular pathogens and viruses alike [32, 33]. Meanwhile, it has reported that *T. gondii* ROP18 is evolved mechanisms to mediate degradation of the host endoplasmic reticulum-localizing transcription factor, ATF6β, to down-regulate T cell-mediated type I adaptive immune responses [39].

In the present study, we detected the expressions of related proteins in the ER stress-mediated apoptosis pathway by Immunoblotting analysis, and the results showed that ROP18 enhanced the expressions of cleaved caspase-12, CHOP and cleaved caspase-3 in the neural cells (Fig. 4), and subsequently increased the apoptosis of the neural cells infected by ROP18 over-expressing parasite (Fig. 2). Moreover, with pretreatment of Z-ATAD-FMK, the protein level of cleaved caspase-12 and caspase-3 were significantly reduced and the apoptotic level of neural cells was down-regulated accordingly (Figs. 3 and 4). Taken together, our data indicate that the virulence factor ROP18 in *T. gondii* is involved in the apoptosis of neural cells through the ER stress pathway. Further studies focusing on ROP18-interacting host factors in ER stress pathway will help us to gain new insights into this pathological process.

## Conclusions

Our findings here highlight that the virulence factor ROP18 in *T. gondii* may contribute to neuronal apoptosis through the ER stress-mediated apoptosis pathway, which may contribute to better understanding the possible mechanism of brain pathology during *T. gondii* infection.
